# Skin Cancer May Delay Onset but Not Progression of Parkinson's Disease: A Nested Case-Control Study

**DOI:** 10.3389/fneur.2020.00406

**Published:** 2020-05-28

**Authors:** Abhimanyu Mahajan, Martina Chirra, Alok K. Dwivedi, Andrea Sturchio, Elizabeth G. Keeling, Luca Marsili, Alberto J. Espay

**Affiliations:** ^1^Department of Neurology, James J. and Joan A. Gardner Center for Parkinson's Disease and Movement Disorders, University of Cincinnati, Cincinnati, OH, United States; ^2^Medical Oncology Unit, Department of Oncology, University of Siena, Siena, Italy; ^3^Division of Hematology-Oncology, Department of Internal Medicine, University of Cincinnati, Cincinnati, OH, United States; ^4^Division of Biostatistics and Epidemiology, Department of Molecular and Translational Medicine, Texas Tech University Health Sciences Center, El Paso, TX, United States

**Keywords:** Parkinson's disease, cancer, Hoehn & Yahr, disease progression, disease milestones

## Abstract

**Objective:** To evaluate the extent to which cancer, a biological opposite to neurodegenerative disorders, may affect the onset and progression of Parkinson's disease (PD).

**Methods:** A nested case-control design in consecutive PD patients with (cases) vs. without (controls) cancer was used to compare time to clinical diagnosis and time to Hoehn & Yahr (H&Y) staging score ≥ 3 as a measure of progression. Further, we compared PD onset and progression between cases with cancer diagnosis before (cancer before PD group) and after (cancer after PD group) PD onset. Independent variables were age at PD onset, motor subscale of the Movement Disorders Society-Unified Parkinson's Disease Rating Scale, sex, cognitive impairment, falls, depression, anxiety, dementia, and autonomic symptoms. Time to H&Y ≥ 3 was determined using Cox proportional hazards, with adjusted results summarized as hazards ratio (HR). Group differences were evaluated using unpaired *t*-test or Fisher's exact test.

**Results:** The clinical PD onset was later in cases vs. controls (median 67.2 vs. 59.8 years; *p* < 0.001), but the adjusted time to H&Y ≥ 3 was similar between groups (HR = 0.67; *p* = 0.13). Skin cancers constituted 75% of all cancers in cases. Amongst skin cancers, compared to controls, cases had an older age at PD onset (67.8 vs. 59.8 years; *p* < 0.001). There was no difference in risk of progression in PD patients with skin cancer compared to controls (HR = 0.54, *p* = 0.09).

**Conclusions:** Cancer, in particular of the skin, may delay the onset but not the progression of PD. Future prospective observational studies are warranted to elucidate the complex interactions between these biologically divergent disorders.

## Introduction

The relationship between cancer and sporadic Parkinson's disease (PD) has recently come under scrutiny based on common epidemiological (e.g., smoking, pesticide, estrogen exposure) and genetic (e.g., CYP2D6 alleles) factors, which suggest convergent mechanisms.

While sporadic PD has been associated with a lower incidence of global cancers, skin cancers (both melanoma and non-melanoma skin cancers), prostate, and breast cancers have been reported with higher prevalence in PD compared to age-matched controls (1, 2).

A Hoehn and Yahr staging score of ≥3 is defined by the presence of postural instability, a major motor-based disability milestone in PD (3–5). Using the time to this milestone as a surrogate of progression, we sought to evaluate the effect of cancers, as disorders characterized by dysregulated cellular proliferation, on the time to symptom onset and to H&Y ≥ 3 in PD, a disorder of dysregulated cellular degeneration.

## Methods

The study protocol was reviewed and approved by the Institutional Review Board (IRB # 2019-0526) at the University of Cincinnati and has therefore been performed in accordance with the ethical standards laid down in the 1964 Declaration of Helsinki and its later amendments.

### Experimental Design and Patient Population

Data for this nested case-control study included patients evaluated at the University of Cincinnati James J. and Joan A. Gardner Center for Parkinson's Disease and Movement Disorders between January 1, 2013, and January 1, 2019. Inclusion criteria for cases were (1) PD diagnosis fulfilling the United Kingdom Brain Bank criteria (6), (2) documented histopathological diagnosis of cancer, (3) at least 6 months of follow up for each patient, and (4) quantification of H&Y score. Exclusion criteria were (1) history of cerebrovascular disease (cerebral ischemic lesions and/or severe heart failure), (2) presentations suggestive of atypical parkinsonism, and (3) fractures in the lower extremities affecting postural assessment or weight bearing. The primary endpoint for onset was age at disease onset and, for progression, was time to reaching H&Y stage ≥3. Controls constituted PD patients without cancer from the same period when the cases were selected, who otherwise met inclusion and exclusion criteria. A 1:1 case-control study nested in a cohort of PD patients was selected to maximize statistical power with a balanced design.

### Data Collection

Two independent raters (A.M. and L.M.) extracted the following demographic data from electronic medical records of all eligible patients: gender, age at PD onset, disease duration at last follow-up (years), number of falls at last visit, H&Y staging, Movement Disorder Society-sponsored revision of the Unified Parkinson's Disease Rating Scale (MDS-UPDRS)-III (7) and the neuropsychological test (MMSE or MoCA) (8, 9). Additionally, for cases, we extracted age at cancer onset, type of histologically diagnosed cancer(s), and time between PD onset and cancer diagnosis in years.

Data on the presence/absence of the following non-motor symptoms were collected from the last recorded visit: depression, anxiety, dysautonomic features (gastrointestinal, cardiovascular/orthostatic hypotension, thermoregulatory, urinary, and sexual symptoms (10)), mild cognitive impairment (MCI), and dementia. Dementia was defined as the presence of deficits in at least two of the five core cognitive domains (attention, memory, executive functions, language, and visuo-spatial function) severe enough to affect daily living activities (11) or as an MMSE or MoCA score <26 (11, 12) plus use of an anti-dementia medication. Postural impairment was defined as the time in years for each patient to reach H&Y stage ≥3 (13, 14).

### Statistical Analysis

The baseline characteristics were compared between groups (cases and controls) using a Fisher's exact test or an unpaired *t*-test or a Wilcoxon rank-sum test depending on the type or distribution of the variable. Those who did not develop H&Y ≥ 3 were considered censored events. Progression-free time to H&Y ≥ 3 was estimated using Kaplan-Meier Curve analysis and compared between cases and controls using a log-rank test. Unadjusted and adjusted effects of the presence of cancer on H&Y ≥ 3 were determined using Cox proportional hazards analysis. Variables included in the model were presence of cancer, MDS-UPDRS-III score, cardiovascular symptoms, sex, MCI, falls, depression, anxiety, dementia, and autonomic symptoms (urinary, gastrointestinal, sexual, and thermoregulatory symptoms). We included a full adjustment model as well as a reduced adjustment model including any variables that were found to be statistically significant from univariate analysis. The results of Cox models were presented using hazards ratios (HR), 95% confidence intervals (CI), and *p*-values.

Given numerous potential confounders, we used a propensity-based matching approach, inverse probability of treatment weighting (IPTW-PS), to balance the several potential confounders between cases and controls. This procedure also allowed us to evaluate the clinical factors associated with the cases compared to controls (see Supplementary Data).

In addition, we matched cases and controls for age at PD onset and sex in a 1:1 ratio using the propensity score matching method with caliper widths of 0.001 to assess potential difference in progression to H&Y ≥ 3. The Cox regression was applied on the matched data after accounting for the clustering effect through robust variance estimate. Further, we compared PD onset and progression between cases with cancer diagnosis at least 2 years prior to PD onset (cancer before PD group) and cancer diagnosis at least 2 years after PD onset (cancer after PD group). A period of 2 years was selected to minimize the overlap between the two disorders.

*P*-values less than or equal to a 5% level of significance was considered to indicate statistically significant results. All statistical analyses were carried out using STATA 15 or SAS 9.4.

## Results

A total of 250 consecutive eligible patients (125 cases, 125 controls) were included in the analyses. During a median follow up of 7 years (range: 0–29 years), 78 (31%) reached H&Y score ≥ 3, at a median time of 16 years (95% CI: 14–18 years). Older age at PD onset (HR = 1.10; *p* < 0.001), depression (HR = 1.65, *p* = 0.05), MCI (HR = 2.58; *p* = 0.005), falls (HR = 2.78, *p* < 0.001), and worse motor symptom severity (HR = 1.02; *p* = 0.024) were associated with an increased risk of reaching H&Y score ≥ 3.

The prevalence of different cancer subtypes is outlined in Supplementary Data, with skin cancers constituting 75% of all cancers. No metastatic cancers were found in our sample of patients with cancer (cases). The median age at PD onset was 67.2 ± 10.2 years and, at cancer diagnosis, 71.7 ± 8.7 years.

### Age at Onset and Group Comparisons

**Figure 1:**
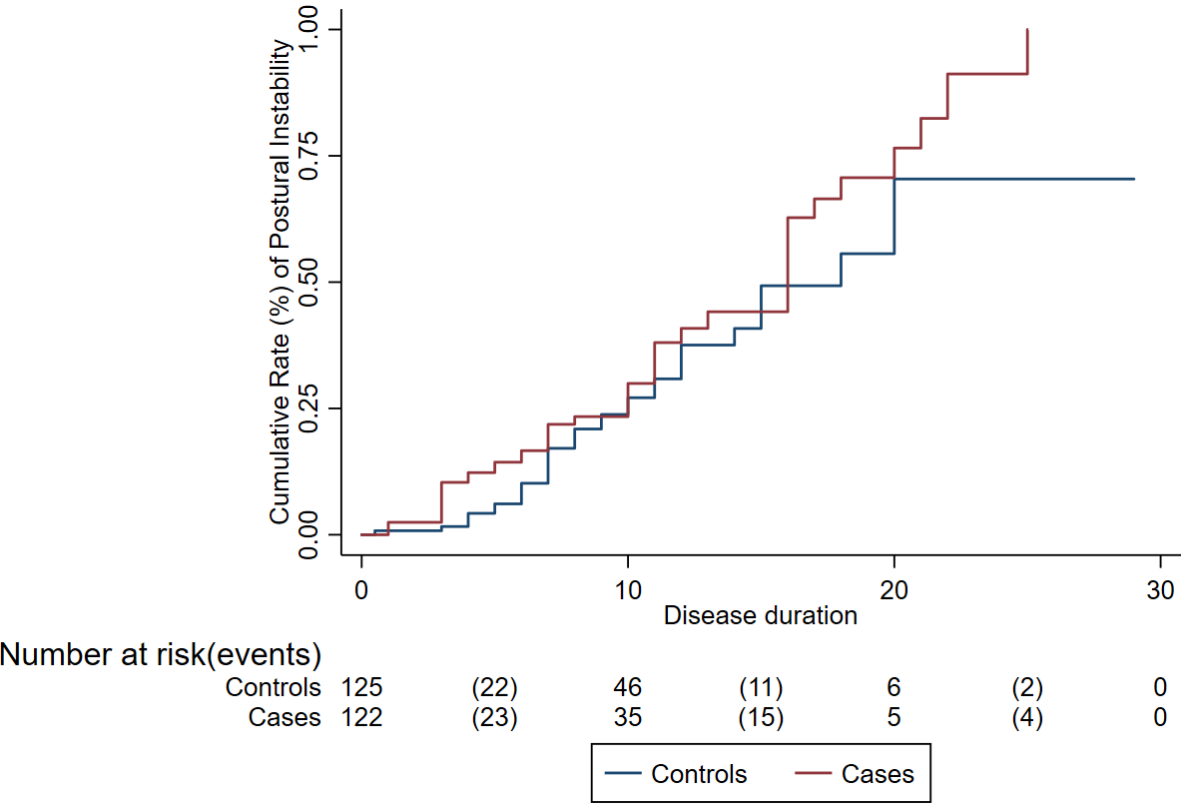
Cumulative rate of disease progression (postural instability) among cases (patients with Parkinson's disease and cancer) and controls (patients with Parkinson's disease only).

Compared to controls, cases had an older median age at PD onset (67.2 vs. 59.8 years; *p* < 0.001). Compared to controls, cases were more commonly male (78.4 vs. 61.6%; *p* = 0.008) and had higher prevalence of MCI (37.2 vs. 24%; *p* = 0.012), dementia (40.8 vs. 26.4%, *p* = 0.010), and urinary symptoms (26.4 vs. 12.8%; *p* = 0.002) but similar prevalence of anxiety (31.6 vs. 40%; *p* = 0.19) and depression (40 vs. 32%; *p* = 0.72) (Table 1). Amongst skin cancers, compared to controls, cases had an older age at PD onset (67.8 vs. 59.8 years; *p* < 0.001). Disease progression. The risk of progression to H&Y score ≥ 3 was not different between cases (30% at 10 years) and controls (27% at 10 years; *p* = 0.18) (Figure 1). After adjusting for potential confounders, the risk of progression to H&Y ≥ 3 was still no different in cases vs. controls (HR = 0.85; 95% CI: 0.46–1.56, *p* = 0.59). There was no difference in risk of progression in PD patients with skin cancer compared to controls (HR = 0.54, *p* = 0.09).

**Table 1:** Comparison of clinical profiles of cases and controls.

**Factor**	**Cases**	**Controls**	***p*-value**
*N*	125	125	
Sex (female)	27 (21.6%)	46 (36.8%)	0.008
Age at PD onset in years, mean (SD)	67.2 (10.2)	59.8 (12.2)	<0.001
Depression at last visit	50 (40.0%)	40 (32.0%)	0.19
Anxiety at last visit	45 (36.0%)	52 (41.6%)	0.44
Hoehn & Yahr stage at baseline <3	95 (76%)	106 (84.8%)	0.38
Hoehn & Yahr stage at last visit <3	67 (53.6%)	83 (66.4%)	0.069
Patients reaching Hoehn & Yahr 3 and above	43 (34%)	35 (28%)	
Time from symptom onset to Hoehn & Yahr ≥ 3, mean (SD)	9 (6.6)	8.9 (4.6)	
MDS-UPDRS-III at last visit, mean (SD)	27.1 (16.2)	26.0 (17.6)	0.61
Falls (at least 1 episode per year)	51 (40.8%)	43 (34.4%)	0.36
Dementia	51 (40.8%)	33 (26.4%)	0.016
Mild cognitive impairment	47 (37.6%)	30 (24.0%)	0.37
Gastrointestinal autonomic symptoms	51 (40.8%)	54 (43.2%)	0.52
Cardiovascular autonomic symptoms	32 (25.6%)	33 (26.4%)	1.00
Thermoregulatory autonomic symptoms	10 (8.0%)	10 (8.0%)	1.00
Urinary autonomic symptoms	33 (26.4%)	16 (12.8%)	0.016
Sexual autonomic symptoms	10 (8.0%)	14 (11.2%)	0.29
Death	11 (8.8%)	7 (5.6%)	0.34
Age at death, mean (SD)	77.5 (8.6)	75.5 (9.6)	0.71

*SD, Standard deviation; IQR, Interquartile range; MDS-UPDRS-III, Motor subscale of the Movement Disorder Society-sponsored revision of the Unified Parkinson's Disease Rating Scale*.

### Propensity Score Matching

Compared to controls (*n* = 64) matched for age at PD onset (mean age 64.9; SD = 9.1 years) and sex (Male: Female: 49: 15), cases (*n* = 64) were not associated with a different risk of progression to H&Y ≥ 3 (HR = 0.84, *p* = 0.53).

### Cancer Before PD vs. Cancer After PD

The cancer-before-PD group (*n* = 20) showed an older age at PD onset (73.6 vs. 65 years, *p* < 0.001), with no difference in age at postural instability as defined by H&Y ≥ 3 (74.6 vs. 75 years; *p* > 0.05).

## Discussion

This first attempt at elucidating the effect of cancer comorbidity on the clinical onset and progression in PD suggests that cancer lengthens the time to the clinical onset of PD but does not affect its clinical progression, as measured by time to H&Y score ≥ 3, a measure of postural instability heralding the onset of falls, a major milestone in PD. Even after matching cases and controls for age at PD onset and sex, this observation was maintained.

Common genetic mechanisms, especially those underlying cell cycle turnover and protein regulation such as those involving *SNCA, PARK2, PARK8, ATM, PTEN, PINK1*, and *MC1R* have been implicated in both neurodegeneration and cancer and perhaps explain this association (15–17). PD-associated genes are involved with a variety of cellular processes, including control of cell cycle, protein misfolding and degradation, and mitochondrial damage, amongst others. Another proposed mechanism includes the accumulation of DNA mutations secondary to chronic inflammation in neurons and tumors (18). Whereas prior studies have assessed the association of cancer and PD risk (19), this is the first analysis to use H&Y score ≥ 3 as a progression surrogate to evaluate the effect of cancer on disease progression in PD. PD patients have previously been associated with a reduced risk of cancer (20). Conversely, cancer patients have been reported to have a lower risk of developing PD, even after controlling for cancer-related lifestyle factors and correcting for survival bias (21). Recently, an inverse relationship between cancer and memory decline was reported in a large population-based cohort of Alzheimer's disease patients (22). Collectively, these studies suggest a potential risk-attenuating effect of cancer on PD.

On further evaluation of this phenomenon in PD patients with co-existent cancer, we found that predating cancer appears to lengthen the time to PD onset, with no effect on the clinical onset of postural instability. The decision to include a 2-year gap between cancer and PD onset was made to minimize (even if it does not entirely exclude) the overlap between the two disorders. The retrospective design of this clinical study otherwise limits the extent to which we can investigate the relative timing of biological onset of cancer or PD, which represents a key scientific question underlying our hypothesis.

The characteristics of the patient population at the University of Cincinnati may be considered representative of the United States population (23), strengthening the validity and generalizability of our results. Although the data demonstrated a more limited diversity of cancer subtypes than what has been published previously from a Florida cohort, the cancer profile after PD symptom onset was similar (24). It is plausible that unaccounted-for environmental factors, including latitude and sun exposure, play a role in this differential prevalence of cancers between cohorts (25, 26).

The absence of genetic data to establish a biological basis for the aforementioned associations and the inability to replicate recently reported cancer risks from PD-associated mutations (27) are major limitations of our study. Other shortcomings are inherent to the retrospective design of this study, including the likelihood of missing relevant data. Recall bias was mitigated by the prospective collection of clinical data. The absence of detailed data on cancer staging, which may have served to determine if there is any “dose effect” of cancer severity on PD symptoms, is another shortcoming. It is plausible that patients undergoing cancer treatment might ignore PD-related symptoms, thus extending the latency of the recognition of the onset of PD. While the number of cases undergoing immune modulation, chemotherapy, and/or radiation in our sample is anticipated to be low due to the predominant cancer subtype, we cannot discount the possibility that some may have undergone treatment and that such treatment influenced the outcome. Although data from a phase 2 clinical trial of nilotinib in PD showed some concerns regarding safety and questionable preliminary evidence of efficacy, the role of immune modulators as a disease-modifying strategy in PD continues to be a topic under active investigation (28, 29). Due to multiple dopaminergic dose changes between clinic visits, we could not evaluate the effect of medications and cancer. Finally, we cannot exclude the possibility that some subjects in the control group may have developed subclinical cancer later in their disease course and represented misclassifications.

In conclusion, cancer (and skin cancer in particular) appears to delay the time to PD onset but not its clinical progression. Future prospective multi-center observational studies with longer follow up, more granular documentation of both cancer types and PD, and collection of genetic data will serve to shed light on the complex mechanisms of interactions between these biologically divergent disorders when concurrently present. Such an effort would also help elucidate the interacting role of environment and genetics and any putative protective role of certain genetic mutations, especially early in the disease.

## Data Availability Statement

Data not published within the article will be made available for analyses upon request. Where patient data can be anonymized, the authors will share the summary tables that underlie the results reported in this article with qualified researchers who provide a valid research question, following a signed data access agreement.

## Ethics Statement

The studies involving human participants were reviewed and approved by University of Cincinnati Institutional Review Board. Written informed consent for participation was not required for this study in accordance with the national legislation and the institutional requirements.

## Author Contributions

AM contributed to the conception, organization, and execution of the study design, the design, review, critique of the statistical analysis, the writing of the first draft of the manuscript and its review, and critique. MC contributed to the organization, execution of the study design, and the review and critique of both the statistical analysis and the manuscript. AD contributed to the organization and execution of the study design, and the review and critique of both the statistical analysis and the manuscript. AS contributed to the execution of the study design and the review and critique of both the statistical analysis and the manuscript. EK contributed to the execution of the study design and the review and critique of the manuscript. LM contributed to the conception, organization, and execution of the study design and the review and critique of both the statistical analysis and the manuscript. AE contributed to the conception and organization of the study design, the review and critique of both the statistical analysis and the manuscript, and gave final approval of the submitted version.

## Conflict of Interest

The authors declare that the research was conducted in the absence of any commercial or financial relationships that could be construed as a potential conflict of interest.
