# Supplementary material for: Skin Cancer May Delay Onset but Not Progression of Parkinson's Disease: A Nested Case-Control Study
Source: Front Neurol. 2020 May 28;11:406. doi: 10.3389/fneur.2020.00406 (PMC7270344; doi:10.3389/fneur.2020.00406)
Supplement: Supplementary file 1 [file Data_Sheet_1.docx]

**Supplementary data**

Three different models were used using IPTW-PS method. Model 1 only included age and gender, model 2 included major baseline factors (age, gender, weight, and MDS-UPDRS III) and model 3 included all considered baseline factors. In addition, each regression model was also developed after adjusting for prognostic factors for H&Y score ≥3. In IPTW-PS method, we first developed propensity scores using logistic regression with baseline variables. After obtaining propensity score, weighted Cox regression model with robust variance estimate was used to evaluate the effect of cases on progression to postural instability compared to controls. Weight was allocated to each subject using inverse probability treatment approach.

**Table 1:** Effect of Cases on Progression to Postural Instability Compared to Controls- Propensity Score Validation analysis

| Model | Group | HR | 95%CI | | p-value |
| --- | --- | --- | --- | --- | --- |
| Model 1a | PD+cancer | 0.82 | 0.51 | 1.31 | 0.404 |
| Model 1b | PD+cancer | 0.81 | 0.46 | 1.41 | 0.45 |
| Model 2a | PD+cancer | 0.82 | 0.50 | 1.34 | 0.426 |
| Model 2b | PD+cancer | 0.81 | 0.46 | 1.42 | 0.46 |
| Model 3a | PD+cancer | 0.83 | 0.48 | 1.42 | 0.495 |
| Model 3b | PD+cancer | 0.84 | 0.51 | 1.39 | 0.49 |

*HR: Hazards ratio; CI: confidence interval

**Model 1:** (a) Inverse probability of treatment weighting (IPTW) using the propensity score (PS) based on age and gender (b) IPTW using the PS based on age and gender and adjusting for prognostic variables (age, gender, weight, MDS-UPDRS, depression, anxiety, dementia, urinary, gastrointestinal, sexual, thermoregulatory, cardiovascular, and mci; Model 2 (a) IPTW using the PS based on age, gender, weight, and UPDRS (b) IPTW using the PS based on age, gender, weight, and MDS-UPDRS and adjusting for prognostic variables; Model 3 (a) IPTW using the PS based on age, gender, weight, MDS-UPDRS, depression, anxiety, dementia, urinary, gastrointestinal, sexual, thermoregulatory, cardiovascular, mci, and falls per year (b) IPTW using the PS based on age, gender, weight, MDS-UPDRS, depression, anxiety, dementia, urinary, gastrointestinal, sexual, thermoregulatory, cardiovascular, mci, and falls per year and adjusting for prognostic variables.

**Table 2:** Balance diagnostics: Comparing baseline cofactors between cases and controls using IPTW-PS method

|  | PD+Cancer | PD | SD |
| --- | --- | --- | --- |
| Model 1 | Mean/% | Mean/% |  |
| Age at disease onset | 63.35 | 63.66 | -0.027 |
| Male | 0.70 | 0.70 | -0.006 |
|  |  |  |  |
| Model 2 |  |  |  |
| Age at disease onset | 63.20 | 63.33 | -0.011 |
| Weight | 183.30 | 182.18 | 0.027 |
| MDS UPDRS | 26.62 | 26.52 | 0.006 |
| Male | 0.70 | 0.70 | -0.004 |
|  |  |  |  |
| Model 3 |  |  |  |
| Age at disease onset | 64.86 | 63.95 | 0.08 |
| Weight | 180.45 | 179.87 | 0.014 |
| MDS UPDRS | 26.92 | 27.21 | -0.017 |
| Male | 0.69 | 0.70 | -0.025 |
| Depression | 0.33 | 0.30 | 0.06 |
| Anxiety | 0.38 | 0.38 | -0.007 |
| Dementia | 0.34 | 0.34 | 0.003 |
| gastrointestinal | 0.45 | 0.47 | -0.03 |
| cardiovascular | 0.27 | 0.25 | 0.056 |
| thermoregulatory | 0.08 | 0.09 | -0.027 |
| urinary | 0.19 | 0.19 | 0.013 |
| falls1episodeperyear | 0.35 | 0.38 | -0.062 |
| sexual | 0.08 | 0.11 | -0.092 |
| MCI | 0.30 | 0.27 | 0.075 |
| MCI-unknown | 0.41 | 0.44 | -0.062 |

*SD: standardized differences

**Table: Prevalence of cancer subtypes in Parkinson’s disease in our study sample**

| **Cancer type** | **Prevalence**  **n = 125 (%)** |
| --- | --- |
| Skin | 94 (75.2%) |
| Basal cell carcinoma | 43 (34.4%) |
| Melanoma | 6 (4.8%) |
| Squamous cell carcinoma | 32 (25.6%) |
| Mixed | 12 (9.6%) |
| Non-specific skin cancer | 1 (0.8%) |
| No type specified | 31 (24.8%) |
| Prostate cancer | 16 (12.8%) |
| Bladder cancer | 3 (2.4%) |
| Acute myeloid leukemia | 1 (0.8%) |
| Chronic lymphocytic leukemia | 3 (2.4%) |
| Thyroid cancer | 3 (2.4%) |
| Adenocarcinoma stomach | 1 (0.8%) |
| Breast cancer | 1 (0.8%) |
| Meningioma | 2 (1.6%) |
| Leg chondrosarcoma | 1 (0.8%) |
| Diffuse large B-cell lymphoma | 1 (0.8%) |
| Endometrial cancer | 1 (0.8%) |
| Hepatocellular carcinoma | 1 (0.8%) |
| Non-small cell lung cancer | 2 (1.6%) |
| Non-Hodgkin lymphoma | 1 (0.8%) |
| Colon cancer | 1 (0.8%) |

Each patient may have more than one type of cancer.
